# The Connection between Cephalic Symptoms and Organic Lesions, in Children

**Published:** 1862-01

**Authors:** B. E. Cotting

**Affiliations:** Roxbury


### "THE CONNECTION BETWEEN CEPHALIC SYMPTOMS AND ORGANIC LESIONS, IN CHILDREN."

*Read before the Norfolk (Mass.) District Medical Society,* November 13th, 1861, by B. E. Cotting, M. D., of Roxbury.*

In repeating the proposition offered for consideration, it will be noticed that the subjects specified are children; the symptoms given, cephalic; the solution sought, the anatomical conditions or changes which underlie or induce the outward manifestations.

If, then, we find a child previously healthy the following *reputed* cephalic symptoms, coming on with marked severity and regularity, viz.:—sudden high fever, ushered in, perhaps, by convulsive agitations; a sharp, frequent pulse; irregular respiration; moaning; staring, injected eyeballs; unusual irritability of temper; great apparent headache, increased by motion, the patient steadying the head by the hands; piercing cries, as of severe and darting pain; great sensitiveness to noise and light; strabismus, or unsteady pupil; great restlessness; twitchings of the face and muscles generally; frequent vomiting, without pain or tenderness in abdomen, often without apparent nausea, and without subsequent relief; constipation more or less obstinate—if we find such a general combination of symptoms, we may have good reason to infer that they are connected with an inflammatory process going on within the cranium, that the pia mater is the principal seat of this inflammation—constituting the disease usually called by the latest writers ACUTE MENINGITIS; and we may also infer that this inflammatory process is accompanied, in the membrane involved, by redness, fulness even to swelling (congestion, so-called)—at first dryness, and then exudatory moisture.

And furthermore, if the disease continues to increase, and (not brought to a rapid termination in severe convulsions) goes on, with varied and irregular intermissions, to loss of perception; paralysis; general muscular relaxation; dilated pupil; stertorous breathing; feeble, fluttering, intermittent pulse;

* It is customary in this Society to propose questions for discussion, and to appoint members (in alphabetical order) to open the discussion by written papers. The following was written in obedience to such a call. Without any special pretensions to originality, the writer, in expressing his own opinions, has not hesitated to avail himself of whatever suited his purpose from others—Reynolds, Wilks, etc., etc.

sunken face; retracted abdomen; involuntary discharges; general prostration, and death—a *post-mortem* examination will show the cerebral membranes more or less intensely injected, but smooth or not granular; the surface of the brain covered in part, or whole, with lymph originating from the membranes; lymph often purulent, sometimes filling the interspaces or conceiling the convolutions by its amount. This lymph is found under the arachnoid, and usually less towards the base of the brain, but in exceptional cases the base seems to have been the seat of the attack, and to be covered with the larger quantity.

Generally little or no change can be discovered in the ventricles, excepting now and then an increased amount of serum. The brain-substance, adjacent to the meningeal inflammation, and evidently disturbed by it as the symptoms indicated, does not always exhibit morbid changes after death. It is sometimes, however, injected or softened. The arachnoid is also occasionally affected, presenting a dry or sticky surface.

This disease is rare. A general practitioner is not likely to see, in his own practice, more than one or two cases in a lifetime. West, with all the advantages of a hospital and a speciality, saw but *seven*, and these were all fatal. The duration of the disease is short—it may be not more than three or four days.

If now, on the other hand, we find in a child of previously unhealthy tendencies, cephalic symptoms somewhat similar to those just enumerated, except, perhaps, that besides being less abrupt and violent in their onset, they are less rapid in their progress—having been preceded for some time by slight febrile disturbances, dull pain in the head, giddiness, uncertain or staggering walk, restlessness, peevishness and the like, most or all of these so light, perhaps, as to have scarcely attracted a passing notice—if, with such antecedents, we find obstinate and unprovoked vomiting, constipation, often a short hacking cough—more or less of these symptoms intermitting at irregular intervals, and recurring with increased severity, with greater pain, wandering delirium, drowsiness, etc.—from intolerance of disturbance to loss of perception, convulsions, hemiplegia, and finally general paralysis—accompanied by a rapid, declining pulse, cold extremities, and at last *death;* if we find such a series progressing in a child which has in itself or through its progenitors a tendency to scrofula, tubercular formations, or other kindred debilitating affections, we may be quite sure that we have to deal with TUBERCULAR MENINGITIS; and that the cephalic symptoms exhibited, whether simple or complicated, arise from the development and growth of tubercular disease in the meninges, communicating a disturbing influence to the cerebral substance.

After death there will be found, in a large majority of cases, lymph at the base of the brain, always tubercles in the pia mater, serous effusion in the ventricles, in a greater degree than in the previously described affection; accompanied usually, though not always, by some perceptible softening or other morbid condition in the adjacent portions of the cerebral substance itself. Oftentimes confirmed tubercular deposits in other organs, especially the lungs, leave no doubt of the true character of the disease.

The duration of the disease is generally two or three, or more, weeks after the symptoms are well confirmed. It is not an unfrequent disease. Every physician is liable to meet with one or more cases every year of his practice. And from what has been said, it may be inferred that it seldom if ever occurs as a primary affection; and that it must of necessity, usually if not always, have a fatal termination.

This is the disease which Wytt (who a hundred years ago gave a very graphic description of it) called *hydrocephalus internus*, from an occasional though by no means constant result of its inflammatory action. He was not so unphilosophical as to give it the absurd title of *acute hydrocephalus*. which has been attributed to him.

Tubercular meningitis is the most common and well-marked encephalic disease in children, and such are the connections between its symptoms and the lesions which observation has revealed.

It is not uncommon for authors to call the diseases we have described meningitis and arachnitis indiscriminately, and to speak of the latter as a frequent affection, but it is our belief that *arachnitis*, uncomplicated, that is, a simple primary inflammation of the arachnoid membrane, is a very rare disease—so rare that some of our most experienced pathologists have never seen a case of it. Whenever adjacent inflammation extends to the arachnoid, it seldom produces any other perceptible lesion than a little greasy or sticky feeling on its surface; and the connection of such lesion with preexisting symptoms is so obscure or so slight as to be of little practical importance. In general those affections of the arachnoid, which become of clinical importance, arise from disease, or external injury, of the dura mater, and are to be studied in connection therewith.

Inflammation of the dura mater, so far as known, is a result of injury or disease externally affecting the bony structures of the head.

Cerebritis.—The "cephalic" symptoms usually ascribed to this affection are dull headache, vertigo, numbness, confusion of thought, defective muscular motion, silliness or want of expression, impaired speech, partial or general paralysis, obscure or partial convulsive movements, rigidity, &c. When such symptoms occur either by themselves or in connection with any other affection, (meningitis, for instance) there is thought to be reason to suspect more or less active inflammation of the brain, and that its substance will be found after death in a disorganized or softened state. But such anticipations are not always confirmed.

Congestion—an early anatomical condition of meningitis—when existing by itself (if ever) may exhibit more transitory symptoms than a confirmed disease. There may be, perchance, less vomiting. Constipation may be accidental. Full pulse and other signs of plethora may be present; and, in general, the obscure indications of congestion may not unfrequently be referred to a distant and perhaps far different affection. But we must not expect tangible *post-mortem* proofs of congestion, as, from the nature of the case, our evidence of its previous existence must be mostly clinical. Dr. Gooch has given evidence to prove that heaviness of the head, and *drowsiness*, attributed "inveterately" to congestion, really depended, in his cases, upon a deficiency of nervous energy; also, that the state of the eye (dilated, motionless) resembling that resulting from *effusion*, as supposed, was in reality due to a deficiency in the circulation of the brain. Marshall Hall and Abercrombie have also described such anæmic cases, arising from derangements of the digestive organs.

Effusion, sometimes called *chronic hydrocephalus* when long a prominent symptom, can hardly be called an organic lesion, although often spoken of as such, but rather the result of a number of various and even opposite pathological conditions. Though the morbid change may be too slight or too obscure to be demonstrated after death in the parts containing the effused liquid, still simple *passive exudation* unpreceded by some such change, is hardly supposable, even on the loosest theory of functional derangement only. As effusion into the chest is now ascribed previous to latent disease, however slight and unobservable its symptoms, so encephalic exudation must have a similar origin, though its occasion may be in a distant locality. Moreover, effusion is often only an attendant upon a moribund state—a mere closing up, it may be, of some remote affection (cholera infantum, for instance) —a mortuary result, not a previous complication.

Special symptoms as indications of specific organic conditions are, we fear, hardly worthy of the reliance often placed in them. *Dilated pupil* as an evidence of effusion; *contracted*, when fluid exists in large quantities at the base of the brain, or into the pons Varolii, are states not permanent in the same individual case, but often alternate from unknown or very slight causes. The *half-closed eye*, covered where exposed with mucoid film, thought by some to be a reliable diagnostic or disease of the brain, we recently found wholly delusive in a very marked instance.

*Hemiplegia* may depend, as asserted, upon a greater softening, or disorganized condition of one side of the cerebrum than the other, but every one, who has tried to prove this, will acknowledge the difficulty.

*Paralysis* may probably be attributed, with reason, to the giving way of the central portions of the brain, as is generally supposed.

Convulsions, on the contrary, are said to have their origin in causes affecting the surface of the brain; but their causes, various and apparently dissimilar as they often appear, may nevertheless produce similar cerebral disturbance, either through direct or reflex movements in the nervous system.

Convulsions are striking symptoms—symptoms only—of diseased changes taking place, perhaps within the cranium near the seat of their origin, or in other and possibly very distant parts of the body. Whatever may be the part of the acting nervous material of the brain-substance, which under an influence which we will call cerebral disturbance, for want of a better term, gives rise to convulsive agitations, it is not unlikely that this peculiar disturbance is brought about whenever any disease is accompanied by convulsions. To this disturbance, and not to any organic lesion necessarily connected with the disease in question, whether such disease be near the brain or far from it, we must ascribe these convulsions. Thus may be accounted for, not unphilosophically, the convulsive movements occurring in gastric as well as in meningitic diseases, and the fact also that these movements are not uniformly nor necessarily attendant upon any of such diseases. Thus, too, may be explained the temporary or paroxysmal characters of convulsions generally.

Taking such views, and remembering the greater nervous development and susceptibility in children than in adults, we may understand why a disorder or a disease, which in adults would ordinarily be ushered by *rigors*, may commence in children with convulsions—the convulsions as well as the rigors not indicating *the* disease, but only the coming on of some affection to be thenceafter made apparent.

So, also, when in the course of any disease, an increase or metastasis is taking place, and we have in adults a repeated rigor or marked delirium, we may expect in children an onset or recurrence of convulsions.

In all cases the convulsions must be considered as consequences of a disturbance in the acting material of the brain, and not the cause of the incoming, changing or rapidly increasing severity of the disease. In the one case they warn us to look out for an approaching or newly-arrived evil, of slight or serious character it may be; while in the other, in the course of any disease, they generally announce impending danger, not from themselves merely, but from an increase, or unfavorable advance, or complication of the affection then in progress.

Of the number and variety of the diseases or morbid conditions which induce or are accompanied by *convulsions*, every one in active practice must had sufficient experience.

Finally and generally, as the functions of the brain must depend essentially upon the peculiarities of the cerebral structure and not upon the membranes, any derangement of function implies a primary or secondary change in the acting nervous material itself. This change may not be any the less organic because we cannot discover it after death; but we should not forget that the symptoms of such change, cerebral symptoms, are not due, as essential elements, to affections or organic lesions in other textures, however near or distant they may be. Inflammations or diseases of the investing membranes of the brain itself may exist without inducing cerebral symptoms, but the connection between the meninges and the brain-substance is so intimate, and their integrity so important, that disease in them is probably more likely to prove the occasion of cerebral derangements, though such are as truly secondary as when arising from other and remoter affections. —*Boston Medical Journal.*

